# Supplementary figures and images for: Phenotyping of a rice (Oryza sativa L.) association panel identifies loci associated with tolerance to low soil fertility on smallholder farm conditions in Madagascar
Source: PLoS One. 2022 May 18;17(5):e0262707. doi: 10.1371/journal.pone.0262707 (PMC9116655; doi:10.1371/journal.pone.0262707)

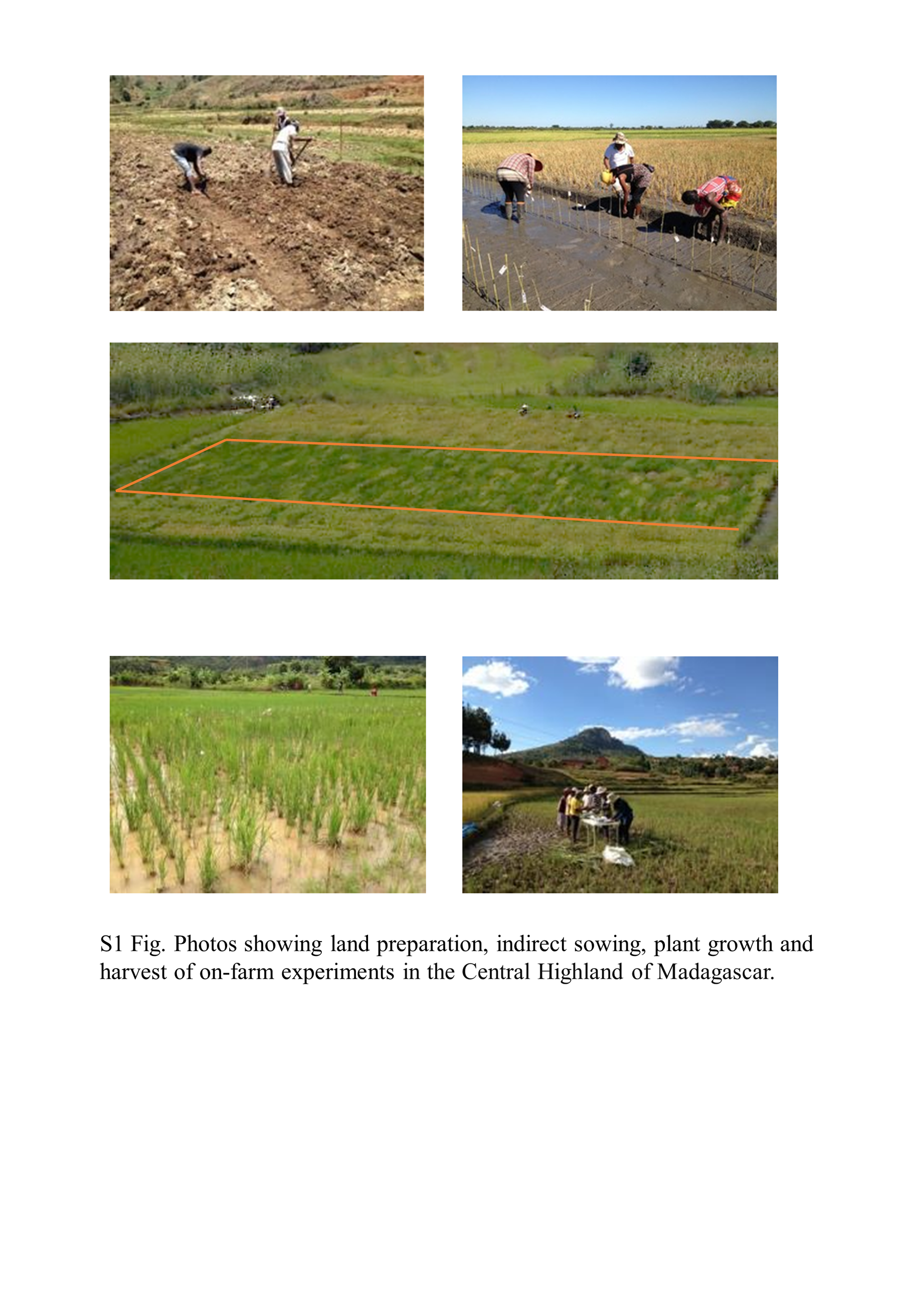

Supplement: S1 Fig — (TIF) [file pone.0262707.s001.tif]

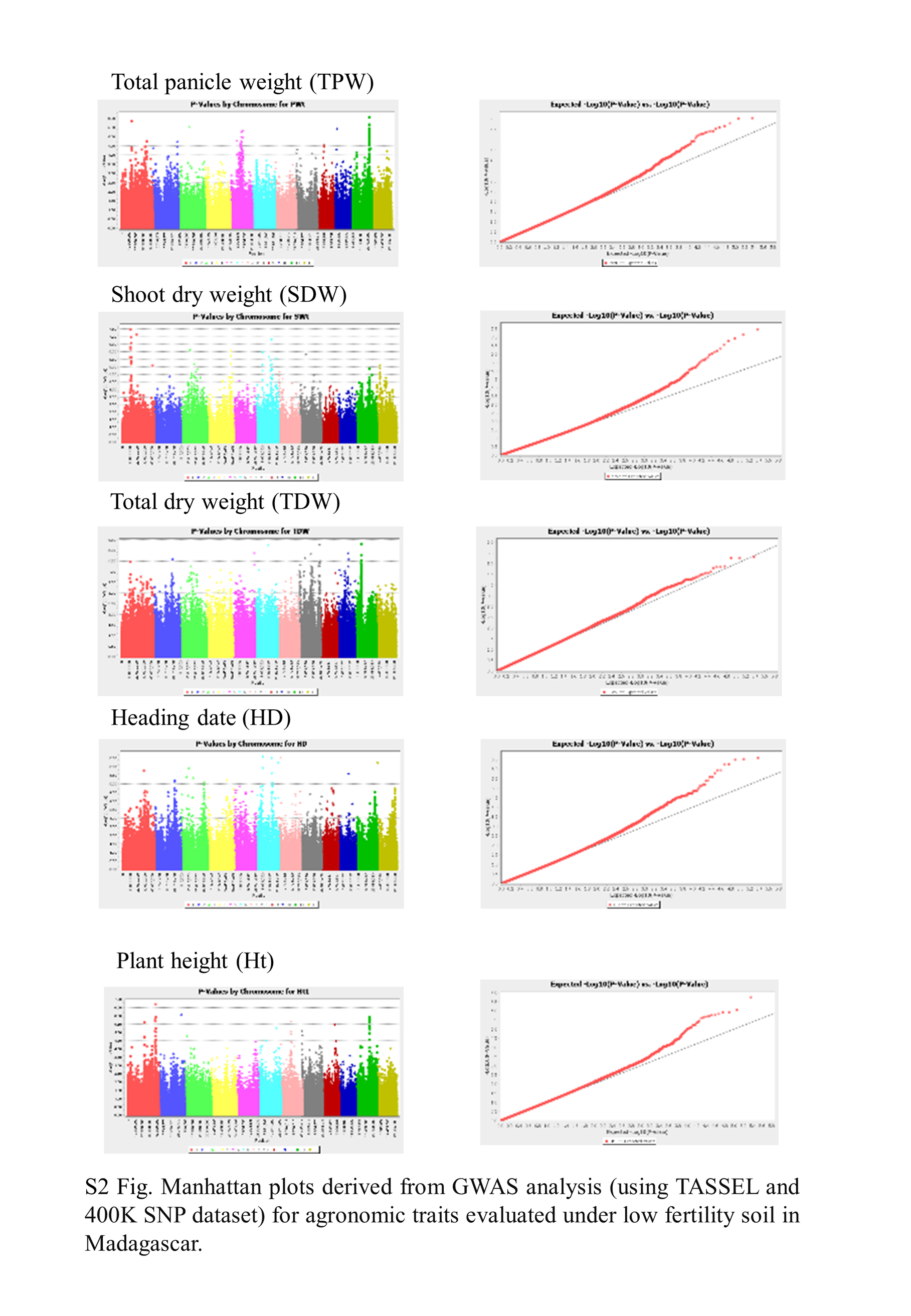

Supplement: S2 Fig — Manhattan plot shows negative logarithmic ((-log10 (P)) values of association for each SNP (Y axis), and SNP location along the 12 chromosomes (colored bar in X axis). Red line indicates a -log10 (P value) threshold of 5. (TIF) [file pone.0262707.s002.tif]

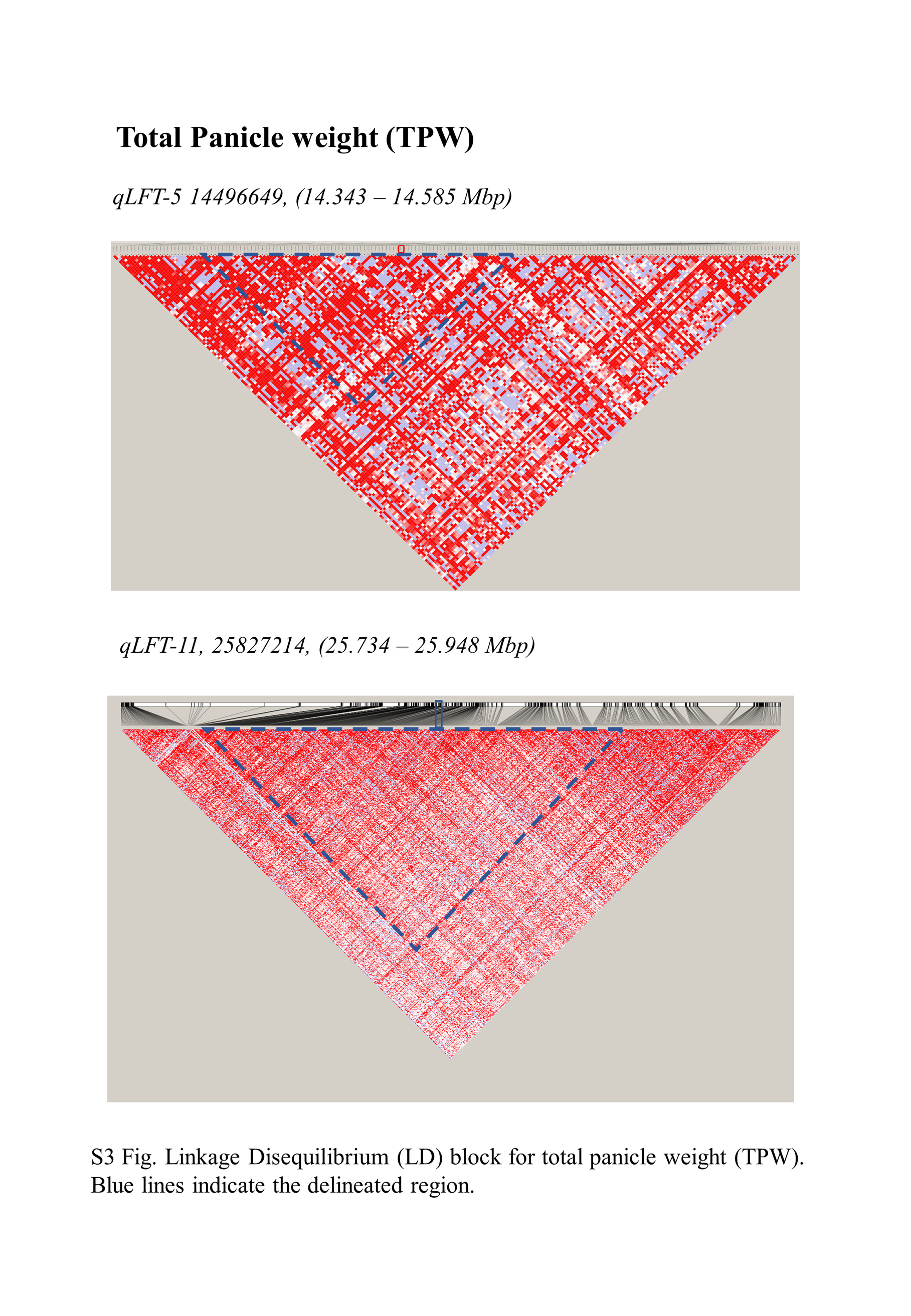

Supplement: S3 Fig — Blue lines indicate the delineated region. (TIF) [file pone.0262707.s003.tif]

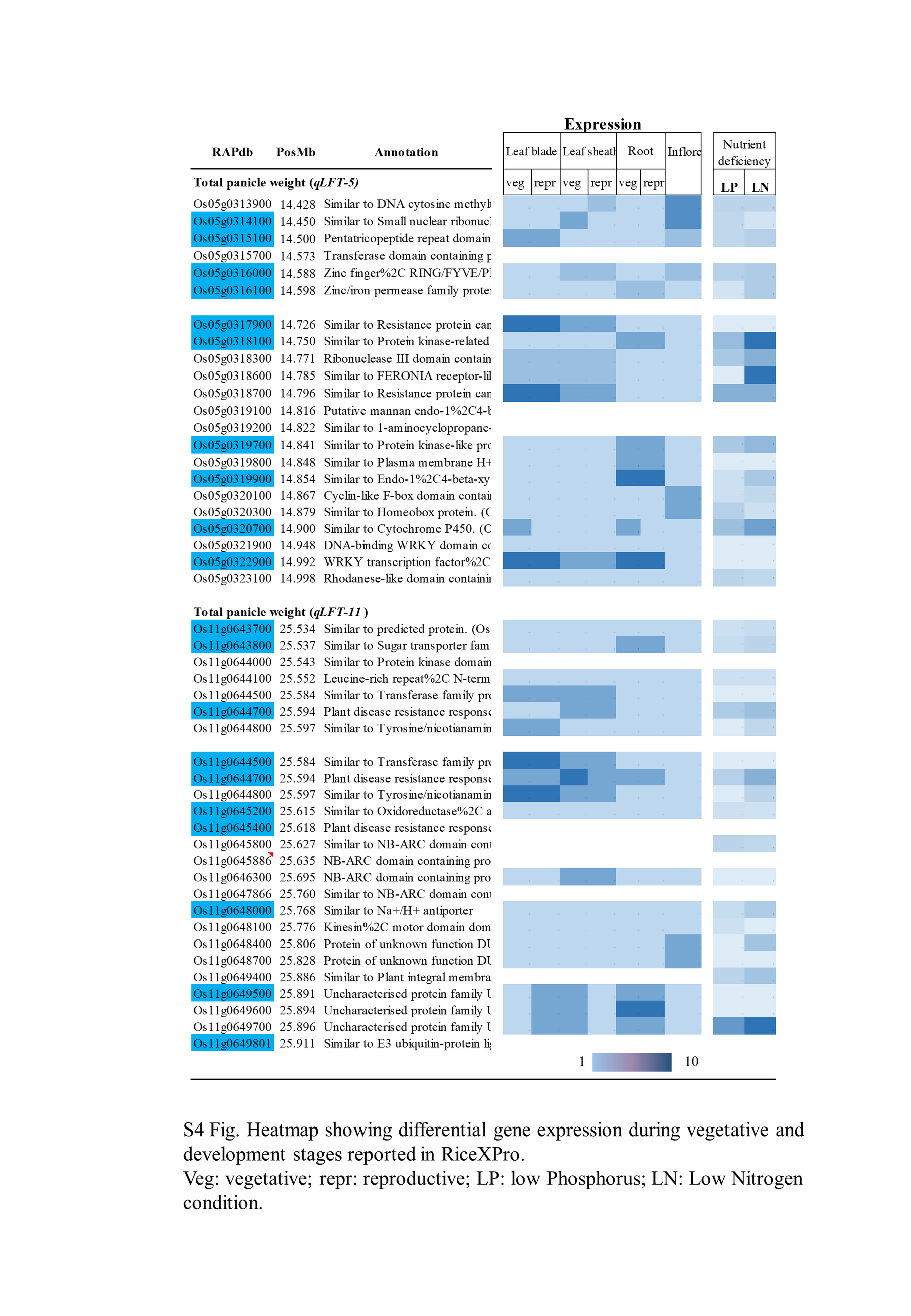

Supplement: S4 Fig — Veg: vegetative; repr: reproductive; LP: low Phosphorus; LN: Low Nitrogen condition. (TIF) [file pone.0262707.s004.tif]

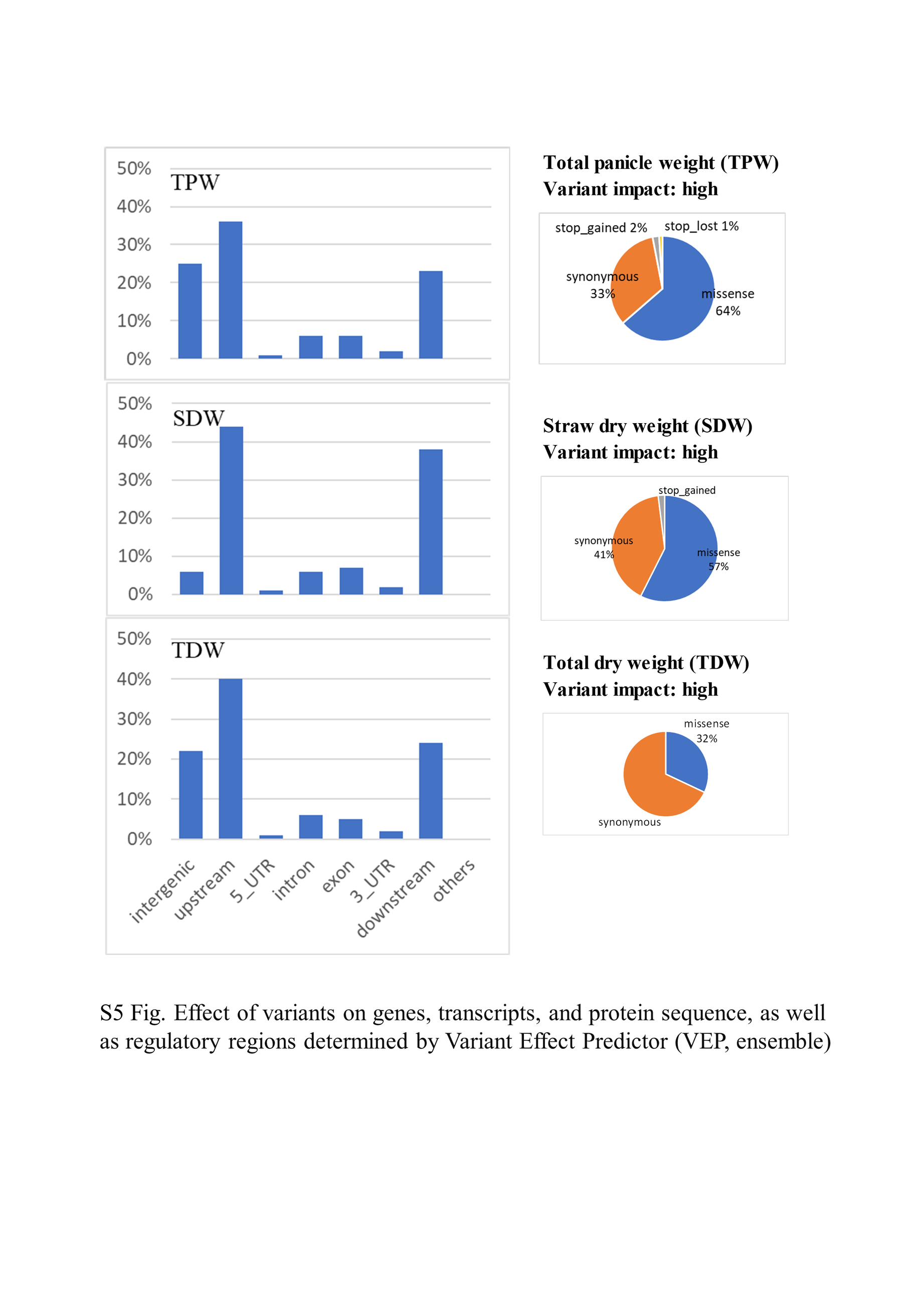

Supplement: S5 Fig — (TIF) [file pone.0262707.s005.tif]
