# Supplementary material for: Phenotyping of a rice (Oryza sativa L.) association panel identifies loci associated with tolerance to low soil fertility on smallholder farm conditions in Madagascar
Source: PLoS One. 2022 May 18;17(5):e0262707. doi: 10.1371/journal.pone.0262707 (PMC9116655; doi:10.1371/journal.pone.0262707)
Supplement: S2 Table — (DOCX) [file pone.0262707.s007.docx]

**S2 Table**. Descriptive statistics and summary of phenotypic traits (Experiment 1)
