# Supplementary material for: Phenotyping of a rice (Oryza sativa L.) association panel identifies loci associated with tolerance to low soil fertility on smallholder farm conditions in Madagascar
Source: PLoS One. 2022 May 18;17(5):e0262707. doi: 10.1371/journal.pone.0262707 (PMC9116655; doi:10.1371/journal.pone.0262707)
Supplement: S4 Table — (DOCX) [file pone.0262707.s009.docx]

**S4 Table**. Allele frequency of total panicle weight (PWT) and straw dry weight (SDW) in the 3K-Rice Genome Project (3KRGP)

ind1, ind2 and ind3: groups of indica rice, and indx: other indica varieties,

temp: temperate, trop: tropical, and japx: other japonica varieties

aus: aus; inax: admixed aus and indica; aro: aromatic; admix: all other unassigned varieties.
