# Supplementary material for: Phenotyping of a rice (Oryza sativa L.) association panel identifies loci associated with tolerance to low soil fertility on smallholder farm conditions in Madagascar
Source: PLoS One. 2022 May 18;17(5):e0262707. doi: 10.1371/journal.pone.0262707 (PMC9116655; doi:10.1371/journal.pone.0262707)
Supplement: S5 Table — (DOCX) [file pone.0262707.s010.docx]

**S5 Table**. Complete list of gene models included in GWAS associated loci
