# Supplementary material for: Phenotyping of a rice (Oryza sativa L.) association panel identifies loci associated with tolerance to low soil fertility on smallholder farm conditions in Madagascar
Source: PLoS One. 2022 May 18;17(5):e0262707. doi: 10.1371/journal.pone.0262707 (PMC9116655; doi:10.1371/journal.pone.0262707)
Supplement: S6 Table — (DOCX) [file pone.0262707.s011.docx]

**S6 Table**. Descriptive statistics and summary of phenotypic traits from the Validation-hydroponic experiment (Experiment 2-2)

|  | Plant height (Ht) | Root length (RL) | Number of roots (Rn) | Number of tillers (Till) | Number of leaves (Ln) | Root dry matter (RDM) | Shoot dry matter (ShDM) | Total dry matter (TotDM) | Root/shoot ratio (r/sh) |  |
| --- | --- | --- | --- | --- | --- | --- | --- | --- | --- | --- |
| Treatment (T) | *** | *** | *** | *** | *** | *** | *** | *** | *** |  |
| HP | 73.7 a | 17.6 d | 48.30 a | 1.2 a | 8.0 a | 0.071 a | 0.367 a | 0.398 a | 0.10 c |  |
| LN | 63.7 b | 35.9 b | 39.53 b | 1.0 b | 5.5 b | 0.071 a | 0.236 b | 0.307 b | 0.29 b |  |
| LP | 61.3 b | 41.1a | 36.20 b | 1.0 b | 5.5 b | 0.071 a | 0.216 b | 0.288 b | 0.33 a |  |
| LNP | 62.4 b | 29.8 c | 37.58 b | 1.0 b | 5.6 b | 0.039 b | 0.196 b | 0.267 b | 0.35 a |  |
|  |  |  |  |  |  |  |  |  |  |  |
| Allele (A) | *** | *** | ns | ns | ns | *** | * | * | *** |  |
| Advantageous | 70.4 a | 35.1 a | 41.0 a | 1.0 a | 6.2 a | 0.071 a | 0.255 a | 0.338 a | 0.29 a |  |
| Disadvantageous | 60.1 b | 27.2 b | 39.7 a | 1.0 a | 6.2 a | 0.054 b | 0.233 a | 0.292 b | 0.24 b |  |
|  |  |  |  |  |  |  |  |  |  |  |
| GxS | ns | ns | ns | ns | ns | ns | ns | ns | ns |  |
|  |  |  |  |  |  |  |  |  |  |  |
| n | 20 | 20 | 20 | 20 | 20 | 20 | 20 | 20 | 20 |  |
| Mean | 64.66 | 30.71 | 40.10 | 1.07 | 6.52 | 0.06 | 0.24 | 0.31 | 0.27 |  |
| SD | 10.87 | 11.19 | 9.03 | 0.21 | 3.65 | 0.02 | 0.08 | 0.08 | 0.10 |  |
| C.V. | 16.8 | 36.4 | 22.5 | 19.3 | 19.0 | 35.6 | 31.4 | 25.5 | 37.8 |  |
| Minimum | 42 | 15 | 27 | 1 | 5 | 0.018 | 0.121 | 0.155 | 0.07 |  |
| Maximum | 90.0 | 63.0 | 77.5 | 2.0 | 10.5 | 0.106 | 0.486 | 0.540 | 0.460 |  |
|  |  |  |  |  |  |  |  |  |  |  |
|  |  |  |  |  |  |  |  |  |  |  |
