# Supplementary material for: Phenotyping of a rice (Oryza sativa L.) association panel identifies loci associated with tolerance to low soil fertility on smallholder farm conditions in Madagascar
Source: PLoS One. 2022 May 18;17(5):e0262707. doi: 10.1371/journal.pone.0262707 (PMC9116655; doi:10.1371/journal.pone.0262707)
Supplement: S7 Table — (DOCX) [file pone.0262707.s012.docx]

**S7 Table**. Allelic distribution between the donor and recipient for the total panicle weight (PWT) QTL.

|  |  |  |  |  |  |  |  |  |  |  |
| --- | --- | --- | --- | --- | --- | --- | --- | --- | --- | --- |
|  | **SNP** | | | | | | | | | |
| Parents | **5@14496649** | | **5@14827473** | | **11@25604919** | | **11@25827214** | | **11@25849659** | |
| **G1103** | **A** | minor | **T** | minor | **T** | minor | **A** | minor | **C** | minor |
| **X265** | **G** | major | **C** | major | **C** | major | **G** | major | **T** | major |
|  |  |  |  |  |  |  |  |  |  |  |
| G1103: IRIS 313-11949 | |  |  |  |  |  |  |  |  |  |
|  |  |  |  |  |  |  |  |  |  |  |
|  |  |  |  |  |  |  |  |  |  |  |
|  | **SNP** | | | | | | | | | |
|  | **5@14496649** | | **5@14827473** | | **11@25604919** | | **11@25827214** | | **11@25849659** | |
| **group** | **A** | **G** | **C** | **T** | **T** | **C** | **A** | **G** | **C** | **T** |
| indx | 24 | 589 | 38 | 566 | 51 | 548 | 52 | 548 | 56 | 547 |
| aus | 0 | 200 | 76 | 120 | 31 | 167 | 0 | 200 | 0 | 198 |
| ind3 | 39 | 432 | 74 | 386 | 44 | 427 | 47 | 422 | 45 | 427 |
| ind2 | 10 | 272 | 25 | 256 | 2 | 280 | 0 | 277 | 0 | 282 |
| temp | 0 | 287 | 0 | 287 | 4 | 279 | 2 | 279 | 4 | 281 |
| ind1A | 27 | 177 | 29 | 161 | 58 | 139 | 68 | 130 | 67 | 131 |
| ind1B | 1 | 213 | 1 | 213 | 43 | 156 | 40 | 157 | 41 | 158 |
| trop | 7 | 364 | 1 | 370 | 0 | 369 | 6 | 364 | 5 | 361 |
| subtrop | 0 | 112 | 0 | 112 | 0 | 112 | 0 | 104 | 0 | 111 |
| admix | 1 | 101 | 8 | 88 | 6 | 92 | 5 | 91 | 5 | 94 |
| aro | 0 | 76 | 1 | 74 | 0 | 76 | 0 | 70 | 0 | 76 |
| japx | 0 | 83 | 0 | 83 | 0 | 83 | 1 | 82 | 1 | 82 |
