# Supplementary material for: Phenotyping of a rice (Oryza sativa L.) association panel identifies loci associated with tolerance to low soil fertility on smallholder farm conditions in Madagascar
Source: PLoS One. 2022 May 18;17(5):e0262707. doi: 10.1371/journal.pone.0262707 (PMC9116655; doi:10.1371/journal.pone.0262707)
Supplement: Attachment — Submitted filename: REview report.docx [file pone.0262707.s014.docx]

The manuscript entitled: Phenotyping of a rice (Oryza sativa) association panel on smallholder farms under low-input conditions in Madagascar identifies loci associated with tolerance to low soil fertility has high significance for the researchers working on rice. Title of the manuscript would be better if the authors can avoid the repetitive word such as low. The authors have defined the objectives of the study very well. Overall the manuscript is well structured, experiments and analyses have performed very well. In abstract section, I suggest authors to include the names of the traits studied and also the names of donor and recipient parent to increase the impact of the manuscript. In this study, 532 rice accessions were study from 3K rice genome project but authors did not mention any selection criteria of these accessions. Therefore, one or two sentences explaining the selection criteria should be added.

Materials and methods section needs further improvement by adding the names of all the studied traits at one place followed by their individual explanation. Authors claim the area under study as low fertility, but without determining the soil properties of the land how it can be referred as low fertility therefore, authors are suggested to incorporate soil nutrients data particularly for N and P.

Please read line no 144 – 145 “A matrix genotype file composed of 186,229 (187K) SNPs and 3026 accessions was prepared and reported in a previous study”

Here the authors are claiming to use an already prepared matrix of 3026 accessions whereas in this study only 532 accession were included therefor it would be better to explain the reasons.

Overall manuscript reads very well. Results are well explained and validated. The results are original to merit publication. Candidate genes/QTLs identified in this study have been well characterized and being used in MAS. Therefore, after incorporating the highlighted minor correction I recommend this manuscript for publication.
